# Sulphate of Alumina as an Antiseptic and Detergent

**Published:** 1843-04-01

**Authors:** M. J. Pennypacker

**Affiliations:** Resident Physician at the Philadelphia Hospital


### SULPHATE OF ALUMINA

### AS AN ANTISEPTIC AND DETERGENT.

By M. J. Pennypacker, M. D.

Resident Physician at the Philadelphia Hospital.

[Communicated by Professor Dunglison.]

Spruce Street, March 26th, 1843.

*My Dear Sir*,—In the preface to the fourth edition of my work entitled "New Remedies," just published, I stated, that since the article *Aluminæ Lales* was written, the sulphate of alumina, at my suggestion, had been subjected to numerous trials in the surgical wards of the Philadelphia Hospital, and had been found a valuable antiseptic and detergent to ulcers,—and farther, that the detailed results of the observations of the resident surgeons on this matter would be published by me hereafter. Since that preface was written, I have received the following communication from Dr. Pennypacker, one of the resident medical attendants, which I beg the favour of you to publish in your journal.

Believe me, my dear sir,

Yours respectfully and truly,

Robley Dunglison.

*Dr. Clymer.*

—

Philadelphia Hospital, March 22d, 1843.

*Dear Doctor*,—Agreeably to your request, I have made inquiry amongst my colleagues — now in charge of the surgical wards of the house — as to the medicinal value of the sulphate of alumina, applied to irritable, sloughing, gangrenous, or indolent sores.

None of them have kept a regular report of the cases in which it has been used, but they concur in stating it to have acted well, as a stimulant, in indolent conditions of the surface; and in many cases to have effected cures where various other remedies had failed to make an impression. Its antiseptic and detergent properties are considered, amongst us, as established; having found it, in no instance, to disappoint our expectations of removing the loathsome aspect of the sore. My colleagues have used it of different strengths; say from ʒijss. up to ℥iij. of the salt, to ℥vi. of water, and have found that a medium strength, regularly applied, has proved most efficacious. When used of the strength of ℥ij. to ℥iij. to the quantity of water above mentioned, they have found it agree well for a time, but it soon becomes too irritating. This accords with my experience in using it.

The following are the cases in which I have tried the salt in question.

Case I. Bed sores over the sacral region of a patient of broken constitution, who had been confined to the dorsal position for several months; the sores were deep, and burrowing in the cellular tissue, and covered with a greenish-yellow adhesive pus, of a very offensive odour. The use of the salt, in the proportion of ℥iss. to ℥vj. of water, removed the fœtor, and cleansed the sore in two days, but did not stimulate. The patient died in about ten days from the first application.

CASE II, was of milk leg, of ten years date. At the time of commencing the use of the salt this leg was large, hard, inelastic, and completely insensible to most applications. At various periods previous to this, pieces of flesh had taken on mortification and come out, leaving always a foul sore difficult to heal. She commenced using the salt in the proportion of ℥ij. to six of water, which had the effect of removing the fœtor, as well as of repressing all tendency to slough; restoring the sensibility in such a degree as to render it necessary to weaken the solution. All the sores were healed in the space of six or eight weeks. This patient is now well, by the aid of the salt and moderate compression.

CASE III, was an ulcer on the gastrocnemius muscle, resulting from a badly treated furunculus. The lotion was used of the same strength as in the preceding cases. It caused the sore to granulate and heal rapidly.

I have also used it as an application to indolent ulcers of long standing, but in only a few cases with marked benefit; but I may possibly attribute my want of success to not having employed the remedy long enough, as the testimony of some of my colleagues goes to prove. In one case, a saturated solution was used for more than two weeks, without any stimulant effects.

In summing up the result of my observations on the properties of this salt, I think I may safely infer that, as an antiseptic, it is prompt and powerful; and that it is a valuable addition to our list of therapeutical agents.

With much regard and esteem,
I am, yours truly,
M. J. PENNYPACKER.

*Robley Dunglison, M. D.*

